# Gene Expression Analysis of the Hepatotoxicant Methapyrilene in Primary Rat Hepatocytes: An Interlaboratory Study

**DOI:** 10.1289/ehp.7915

**Published:** 2005-08-12

**Authors:** Johanna M. Beekman, Franziska Boess, Heinrich Hildebrand, Arno Kalkuhl, Laura Suter

**Affiliations:** 1 Schering AG, Berlin, Germany; 2 F. Hoffmann-La Roche Ltd., Basel, Switzerland; 3 Bayer HealthCare AG, Wuppertal, Germany; 4 Boehringer Ingelheim Pharma GmbH & Co. KG, Biberach, Germany

**Keywords:** hepatotoxicity, interlaboratory study, methapyrilene, microarray, rat hepatocytes, toxicogenomics

## Abstract

Genomics technologies are used in several disciplines, including toxicology. However, these technologies are relatively new, and their applications require further investigations. When investigators apply these technologies to *in vitro* experiments, two major issues need to be clarified: *a*) can *in vitro* toxicity studies, in combination with genomics analyses, be used to predict the toxicity of a compound; and *b*) are the generated toxicogenomics data reproducible between laboratories? These questions were addressed by an interlaboratory study with laboratories of four pharmaceutical companies. We evaluated gene expression patterns from cultured rat primary hepatocytes after a 24-hr incubation with methapyrilene (MP). Extensive data analysis showed that comparison of genomics data from different sources is complex because both experimental and statistical variability are important confounding factors. However, appropriate statistical tools allowed us to use gene expression profiles to distinguish high-dose–treated cells from vehicle-treated cells. Moreover, we correctly identified MP in an independently generated *in vitro* database, underlining that *in vitro* toxicogenomics could be a predictive tool for toxicity. From a mechanistic point of view, despite the observed site-to-site variability, there was good concordance regarding the affected biologic processes. Several subsets of regulated genes were obtained by analyzing the data sets with one method or using different statistical analysis methods. The identified genes are involved in cellular processes that are associated to the exposure of primary hepatocytes to MP. Whether they are specific for MP and are cause or consequence of the toxicity requires further investigations.

In the last decade, genomic technologies have become gradually integrated into several phases of drug development. In the field of toxicology, drug safety laboratories have begun to use these technologies to assist research to conduct toxicity evaluations on as many potential lead compounds as feasible and to gain a better understanding of the mechanisms of toxicities. For investigators to be successful in the selection of compounds most likely to succeed during preclinical development, the methods they use should have a medium throughput, a short turnaround time, a good predictivity, and be reproducible.

*In vitro* systems are being used in toxicology studies to determine several kinds of toxicities. Mouse lymphoma cells, primary rat hepatocytes, and human lymphocytes are among the mammalian cell systems used to determine mutagenicity (Kilbey et al. 1984). Primary rat or human hepatocytes are used to determine cytotoxicity as well as metabolism of compounds or their ability to induce cytochrome P450 genes (Gómez-Lechón et al. 1988; Paillard et al. 1999). However, only a few laboratories have investigated whether *in vitro* systems can be used in the toxicogenomics evaluation of development compounds. Harries et al. (2001) used the human liver HepG2 cell line to investigate gene expression changes of two hepatotoxins. The results strongly suggested that different mechanisms of hepatotoxicity may be associated with specific markers of gene expression. Waring et al. (2001) showed that gene expression profiles for compounds with similar mechanisms of toxicity tested *in vitro* on primary rat hepatocytes formed clusters, suggesting a similar effect on transcription. Conversely, Boess et al. (2003) characterized several hepatic *in vitro* systems on the basis of gene expression profiling and concluded that the results were poorly comparable with the *in vivo* outcome, depending on the cell culture system used. It is therefore essential to obtain more knowledge on the *in vitro* system used to achieve better understanding and interpretation of genomics data.

As genomics technologies have been introduced more and more in toxicology, the International Life Sciences Institute Health and Environmental Sciences Institute (ILSI/HESI) has formed a consortium with more than 30 pharmaceutical companies to address the issues of reliability and reproducibility of these assays (Robinson et al. 2003). Within the ILSI/HESI consortium, the hepatotoxicity working group evaluated the two hepatotoxicants methapyrilene (MP) and clofibrate by gene expression analysis of rat livers (Baker et al. 2004; Chu et al. 2004; Hamadeh et al. 2002; Pennie et al. 2004; Ulrich et al. 2004; Waring et al. 2004). The results of these studies showed that the transferability of microarray technologies between laboratories posed serious protocol-related issues that could be solved only with appropriate and sophisticated statistical tools (Waring et al. 2004).

In the present study, a toxicogenomics experiment using primary rat hepatocytes was performed in the laboratories of four pharmaceutical companies: Bayer HealthCare AG (BA), Boehringer Ingelheim Pharma GmbH & Co. KG (BI), F. Hoffmann-La Roche Ltd. (RO), and Schering AG (SAG). The cell cultures were exposed to two concentrations of MP, an H1 histamine receptor antagonist (Noguchi et al. 1992) that is known to cause periportal cell necrosis (Steinmetz et al. 1988) and liver tumors in rats (Liijnski et al. 1980; Mirsalis 1987). The study was designed to assess the biologic and experimental variability of the *in vitro* systems of the laboratories, to compare their statistical analysis strategies, and to determine whether an *in vitro* toxicogenomics experiment, performed in different laboratories from cell culture to data analysis, would identify a toxic compound with the same reliability.

To reduce the experimental variability, a cell culture protocol with a standardization of the main parameters such as culture medium was used. However, many steps, including perfusion and RNA isolation, followed the individual in-house protocols. Each laboratory performed Affymetrix gene expression analysis on the RG-U34A chip and analyzed the data according to its own methods/software.

## Materials and Methods

### Test article and formulation.

Methapyrilene hydrochloride (CAS no. 135-23-9, lot no. 037F0929) was obtained from Sigma Chemical Corp. (St. Louis, MO, USA). MP was formulated in dimethyl sulfoxide (DMSO).

### Primary rat hepatocytes.

Primary rat hepatocytes were isolated from 10- to 12-week-old male Han:WIST rats (200–300 g body weight; SAG: Tierzucht Schoenwalde GmbH, Schoenwalde, Germany; BA: Harlan Winkelmann, Borchen, Germany; BI: Charles River Deutsch-land GmbH, Sulzfeld, Germany; RO: RCC Ltd., Itingen, Schweiz) by a two-step collagenase liver perfusion method (Seglen 1972). After perfusion the liver was excised and the cells were resuspended in William’s E medium (WME) without phenol red and filtered. Dead cells were removed by a Percoll (Sigma) centrifugation step (Percoll density, 1.06 g/mL, 50 g, 10 min; only at RO and SAG). Primary hepatocyte viability was assessed by trypan blue exclusion and ranged between 72 and 92% (Table 1).

Cells were cultured in six-well plates coated with collagen (Menal GmbH, Herbolzheim, Germany) at a density of 10^6^ cells/well in 2 mL WME supplemented with 10% fetal calf serum (Invitrogen Technologies, Paisley, UK), glutamine (2 mM), hydrocortisone (54 ng/mL), glucagon (7 ng/mL), insulin (5 μg/mL), penicillin (100 U/mL), streptomycin (100 mg/mL), and gentamicin (10 μg/mL) at 37°C in an atmosphere of 5% CO_2_/95% air. After an attachment period of 3 hr, the medium was replaced by 2 mL serum-free WME, with the same supplements.

### Treatment conditions.

To determine the concentration of MP that causes a toxic response in hepatocytes, each laboratory performed two-dose finding studies. After an overnight preculture period of 16–18 hr, the cells were treated with MP, 0–300 μM (BA and RO), and 0–1,000 μM (BI and SAG) in 0.2% DMSO (final concentration) or vehicle (0.2% DMSO, final concentration). The same procedure was performed for the main study, using the two selected concentrations.

### Biochemistry.

Cytotoxicity was determined as lactate dehydrogenase (LDH) release into the cell culture medium. LDH activity was determined spectrophotometrically with commercially available test kits (Table 1). Enzyme activity in the medium was determined and expressed as percentage of LDH activty present in the medium of vehicle-treated cells.

### RNA isolation.

Cells were harvested at 24 hr after treatment either in Qiagen lysis buffer (RNeasy mini kits; Qiagen, Hilden, Germany) without (BA and SAG) or with proteinase K (BI) or in RNAzol/Bio101 (RO) (RNAzol: Tel-Test, Inc., Friendswood, TX, USA; Bio101: Buena Vista, CA, USA). Total RNA was isolated using Qiagen RNeasy columns. The quality of the RNA was determined using the Agilent Bioanalyzer (Agilent Technologies, Palo Alto, CA, USA). Amounts of RNA were determined with RiboGreen (Molecular Probes, Leiden, the Netherlands) or by OD_260_/OD_280_ determination.

### DNA microarray analysis.

Processing of RNA and microarray experiments were carried out basically as recommended by Affymetrix (Affymetrix, Inc., High Wycombe, UK) (Lockhart et al. 1996), with some user-specific variations (Table 1). Labeled *in vitro* transcripts (10–20 μg) for each RNA sample were hybridized on the RG-U34A array. A starting amount of 5–20 μg total RNA was used for the synthesis of double-stranded cDNA with a commercially available kit (Superscript Choice System; Invitrogen Life Technologies) in the presence of a T7-(dT)_24_ DNA oligonucleotide primer. The cDNA was purified by phenol/chloroform/isoamyl alcohol extraction and ethanol precipitation or using the Affymetrix cleanup columns. The purified cDNA was then transcribed *in vitro* (Enzo Diagnostics, Inc., Farmingdale, NY, USA; Ambion, Inc., Austin, TX, USA) in the presence of biotinylated ribonucleotides to form biotin-labeled cRNA. The labeled cRNA was purified on an affinity resin (RNeasy, Qiagen, or Affymetrix cleanup), quantified, and fragmented. Labeled cRNA (10–20 μg) was hybridized for approximately 16 hr at 45°C onto the RG-U34A array. The arrays were washed and stained with streptavidin-R-phycoerythrin (SAPE, Molecular Probes, CA, USA), and the signal was amplified using a biotinylated goat anti-streptavidin antibody (Vector Laboratories, Burlingame, CA, USA) followed by a final staining with SAPE. Arrays were stained using the GeneChip Fluidics Workstation 400 (Affymetrix). The arrays were then scanned using a confocal laser scanner (GeneArray Scanner 2500; Hewlett Packard, Palo Alto, CA, USA, or Agilent Technologies) resulting in an image file (*.DAT file). Using the Affymetrix software, *.CEL files were calculated from the image files.

### Data analysis.

The *.DAT and *.CEL files were distributed among the participants. The data were condensed and normalized (Table 1). The individual analysis strategy of the raw data is described below.

Investigators at BA identified the genes that are regulated to a statistically significant extent by performing a *t*-test (Welch’s modification; Welch 1938) between the control group and each of the treatment groups using Expressionist software (GeneData, Basel, Switzerland). A *p*-value of 0.01 was chosen in conjunction with a 1.5-fold change cutoff.

Investigators at BI, in addition to the values derived from Microarray Analysis Software (MAS, version 5.0; Affymetrix), performed analysis calculations using the Statistical Analysis System (SAS) software (version 6.12; SAS Institute, Cary, NC, USA). To extract differential expressed genes, the following cutoff criteria were defined. The extracted genes must have a *p*-value of 0.05 (one-sided) according to the Mann-Whitney *U*-test. In addition, each probe set (gene) with a fold change value of at least 1.2 was selected. This approach was used as a first filter (and not considered statistically significant). The generated data can then be analyzed by using in-house marker genes [selected in earlier studies of a licensed database (DB)] or in-depth analysis of single selected genes.

Investigators at RO compared treated and control groups and statistical analyses were performed with in-house developed software. Gene expression changes are measured by the Affymetrix software as fluorescence intensities with a given signal (numerical value) and a qualifier or call (present, absent, marginal). If probe sets are detected as expressed, the call is set to 1; if the probe set is absent, this value is set to 0, and if marginal to 0.5. To allow comparability between microarrays, the signal is scaled using the mean intensity of all probe sets on a chip. The numerical values for several replicates are condensed by using the mean and the SD. Differences in expression levels are expressed as change factors (CHGF), which report the change in expression (signal) between two experimental conditions (baseline = control and treated). If an increase is seen, CHGF is calculated as [(signal treated/signal control) − 1]; for a decrease it is [− (signal control/signal treated) + 1]. Thus, the data are symmetrically distributed around 0; a 2-fold increase gives a CHGF of 1, whereas a 50% reduction gives a CHGF of −1. Statistical analysis was based on analysis of variance and Student’s *t*-test. Gene probes considered “expressed” in 50% of the samples (call ≥ 0.5) and showing fold changes > 1.25 or < −1.25 with a significance value of at least 0.1 (paired *t*-test) in one of the individual data sets were selected.

Investigators at SAG, compared treated and control groups, and statistical analyses were performed with Expressionist software. To extract differentially expressed genes, a *t*-test was used. Genes with a *p*-value < 0.01 and a fold change > 1.5 were extracted from every participant’s experiment set of three.

### *Comparison with an* in vitro *toxicogenomics database.*

The data sets processed by RO were compared with the Roche proprietary *in vitro* toxicogenomics DB consisting of 17 compounds that had been tested previously in at least two concentrations. These compounds were tested following Roche-specific cell culture protocols, which were similar but not identical to the protocol described here. Among them was a previous experiment with MP on rat primary hepatocytes at two concentrations (MP_DB; 100 and 300 μM). The comparisons are based on the individual gene expression ratios (fold changes).

## Results

### Biochemistry.

**Figure 1:**
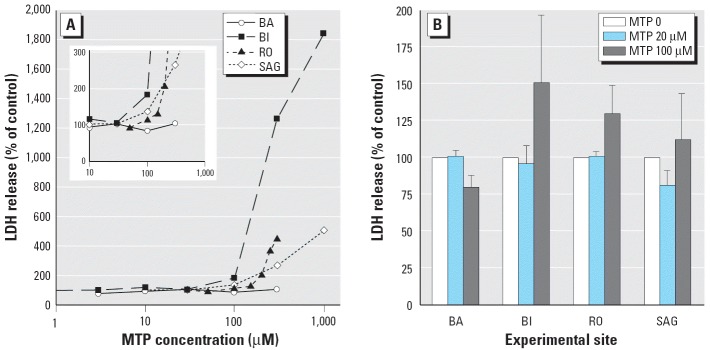
LDH release in the culture medium. (*A*) Pilot study. (*B*) Main study (*n* = 3). Inset in *A* shows the increase in LDH release at 100 μm MP.

In a pilot study the four different laboratories performed a cell culture experiment by incubating primary rat hepatocytes with several concentrations of MP (0–1,000 μM) and analyzing liver enzyme (LDH) release into the medium 24 hr after treatment. Of the four companies, three showed a slight but significant increase of LDH release into the medium at a concentration of 100 μM MP, whereas at a lower dose (20 μM) there was no enhanced LDH leakage compared with untreated cultures (Figure 1A). On the basis of this result, investigators chose a high dose of 100 μM and a low dose of 20 μM for the toxicogenomics experiments.

As anticipated from the results of the pilot experiments, a tendency toward increased LDH release was seen after 24-hr treatment with 100 μM MP during the toxicogenomics experiment (Figure 1B). However, in agreement with the pilot experiment (Figure 1A), this was not seen in all companies. It is important to note that the absolute values of LDH release in the vehicle controls varied considerably between the individual repeats within as well as between the companies, depending on the respective batch of freshly isolated hepatocytes and the different methodologies used to measure the LDH. Therefore, the results were expressed as percentage of LDH release in vehicle-treated cells.

### Gene expression—comparisons across users.

In the toxicogenomics experiment rat primary hepatocytes were incubated with 0, 20, or 100 μM of MP for 24 hr and analyzed for gene expression responses using Affymetrix GeneChips. The raw data (*.CEL and *.DAT files) were exchanged among the participants of this study for individual analysis.

#### Analysis of all data sets with one method.

**Figure 2:**
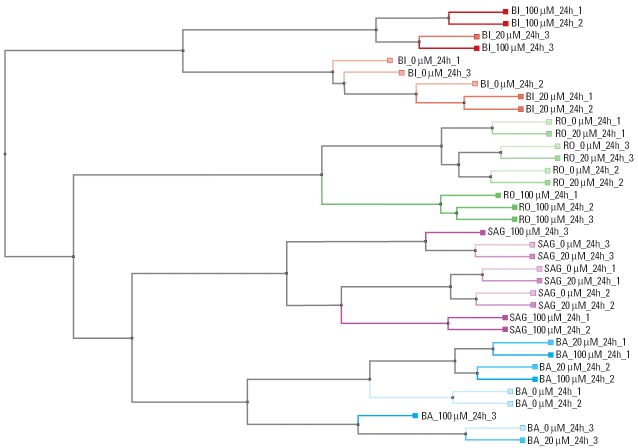
One-dimensional hierarchical clustering of all experiments using all genes of the RG-U34A GeneChip. Distance metric used: positive correlation.

All data sets were analyzed following the analysis strategy from SAG. First, to obtain a general overview of similarities among experimental data sets, a one-dimensional hierarchical clustering (Figure 2) was performed on all data sets. This analysis shows that the data sets cluster together according to their origin. The differences in the gene expression responses are greater between different laboratories than between treated and control hepatocytes.

**Figure 3:**
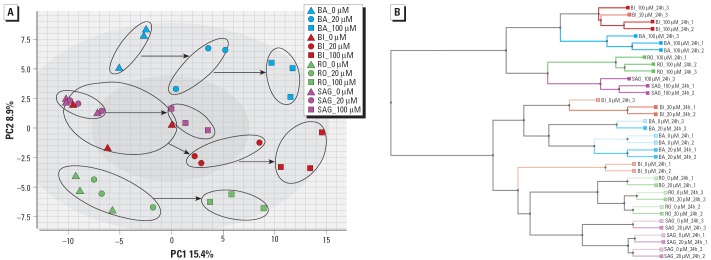
(*A*) PCA of all experiments using the union of genes regulated by MP according to the method of SAG (744 probe sets). Distance metric used: covariance matrix. (*B*) One-dimensional hierarchical clustering of all experiments using the union of genes regulated by MP according to the method of SAG (744 probe sets). Distance metric used: positive correlation.

In the next round of analyses, SAG identified differentially regulated probe sets for each of the participating laboratories (*t*-test with *p* < 0.01 plus fold change > 1.5). This approach eliminates the variability caused by different analysis strategies and reveals the variability due to hepatocyte culture and chip processing protocols. In all studies a substantial increase in regulated probe sets is seen when the MP dose is increased (data not shown). The data set generated from the BI study appeared to have significantly more differentially regulated probe sets at the low dose compared with the other laboratories, whereas the data set of SAG showed the fewest changed probe sets at the high dose. The union of all differentially expressed probe sets results in a number of 744. The overlapping number of probe sets detected as regulated in the experiments of all four users was only five and in at least three of four experiments was 46 (data not shown). The highest concordance between two companies, defined as percentage of “own” genes shared with another company, was 34% (data not shown). When using all 744 probe sets detected as regulated in a principal component analysis (PCA), a distinct separation can be achieved between the untreated samples and those treated with the high-dose MP (Figure 3A). This is in good agreement with the biochemistry data, which showed that slight cytotoxicity was observed at the highest dose of MP, at least by most of the companies. The low-dose samples do not separate well from the untreated for all laboratories. This low dose was chosen as a dose that would not show toxicity based on LDH release. The data show that PC1 (accounting for 15.4% of the variance) drives the treatment-related differences as indicated by the arrows, whereas PC2 (accounting for 8.9% of the variance) shows a separation of the individual laboratories.

The same group of probe sets was used in an unsupervised clustering method, hierarchical clustering. The dendrogram (Figure 3B) shows a clustering of the low-dose samples with their untreated counterparts as well as a clustering of the high-dose samples. The only exception is one of the low-dose samples of BI that clusters together with the high-dose sample of the same experiment.

#### Analysis of one data set with different methods.

**Figure 4:**
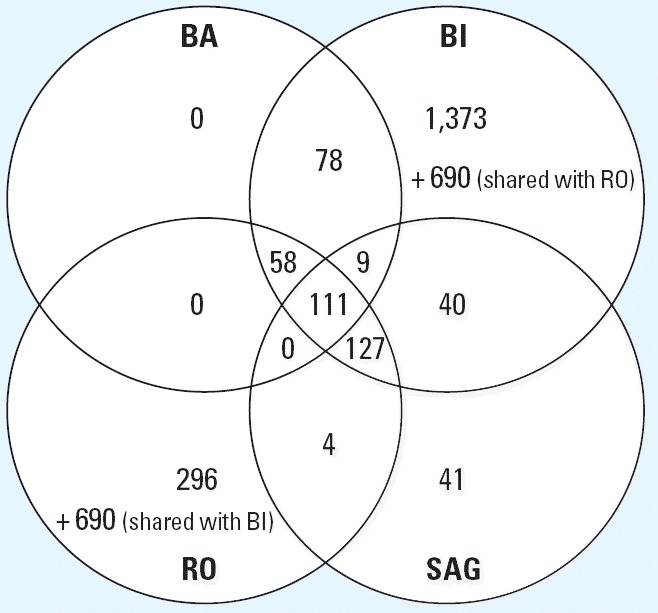
Venn diagram depicting the differentially expressed genes of the BI experiments determined by the four different analysis strategies.

The four laboratories used very different analysis approaches with different main objectives (described in “Materials and Methods” and Table 2). To evaluate the differences of the resulting gene lists generated by the analysis method, the four participating laboratories analyzed one data set (*.DAT or *.CEL files provided by BI) according to their own standard methods. The methods used basically selected genes according to *p*-values from a given statistical test and fold changes (Table 2). RO and BI used a relatively low stringency to select a high number of differentially regulated genes, which then can be compared with their gene expression DB to search for similarities with known toxic compounds. BA and SAG used methods with a higher stringency to obtain gene lists with a low number of false positives. The resulting genes are then annotated and assigned to pathways to determine their biologic significance with respect to the mechanism of toxicity of the investigated compound. Table 2 lists the number of genes found with each method, and Figure 4 displays a Venn diagram depicting the number of genes shared between the different analysis methods. As expected, the different analysis strategies have an immense impact on the number of genes that are defined as differentially regulated. A total of 111 genes were detected with all four methods, whereas three of four methods detected an additional 194 genes (i.e., at least three of four methods detected 305 genes).

#### Analysis of each data set with individual methods.

**Figure 5:**
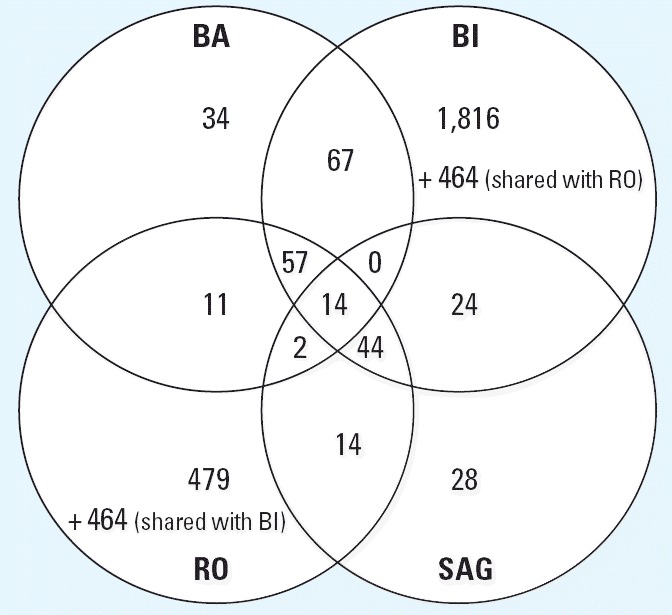
Venn diagram depicting the differentially expressed genes of each company’s experiments determined by its own analysis strategy.

Each laboratory analyzed its own data set using the specific methods as described in “Materials and Methods.” The resulting lists of differentially expressed genes are given in Table 3. Again, as expected, more stringent criteria used by BA and SAG detected only 126 and 185 probe sets as changed, respectively; whereas BI and RO obtained 2,486 and 1,085 probe sets, respectively. Comparison of the gene lists resulting from these analyses shows that BA and SAG share 45% or more of their changed probe sets with BI and RO but only 9–16% with each other. The Venn diagram in Figure 5 shows the relation between the different gene lists. Fourteen genes were detected as regulated by all companies, and an additional 103 genes by three of four companies. The identity of the regulated genes as well as the affected cellular pathways and their biologic significance were determined (Table 4**)**. The probe sets consistently detected by all involved users are associated with detoxification, mitochondrial function, energy production, cell stress, and many general housekeeping processes.

#### Comparison with a gene expression database.

The gene expression profiles of the high-and low-dose MP from the experiments performed in the individual companies (*.DAT files) and analyzed with the strategy of RO were compared with the Roche *in vitro* toxicogenomics DB. At the time of analysis, this proprietary DB contained 47 data sets from 17 different hepatotoxic compounds. The comparison revealed that the high-dose data of each company, except those of SAG, fitted best to the Roche MP data, which were generated in a previous, independent experiment (Table 5). The high dose of SAG and the low doses of all companies were more difficult to predict. When the data sets of this study were incorporated in the DB, the MP data from each company always fitt best to the data from this experiment of the other companies. In most cases, this was also true for the low-dose experiments (Table 5).

## Discussion

The aim of this multisite experiment was to obtain an estimate of lab-to-lab variability for *in vitro* gene expression analysis and to determine whether an *in vitro* toxicogenomics experiment performed in different laboratories from cell culture to data analysis would identify a toxic compound with the same reliability. The toxicogenomics *in vitro* approach shows the known advantages of other *in vitro* test systems, namely, the reduction of the number of animals used for biologic assays as well as the time involved and the cost of the assays. For this investigation, we selected the well-known nongenotoxic hepatocarcinogen MP, which had earlier been chosen as a model hepatotoxin within the ILSI/HESI consortium. To comply with minimal statistical requirements (Lee et al. 2000), each experiment was performed in triplicate using three different batches of primary rat hepatocytes. The number of replicates required to achieve the necessary statistical power was not addressed in this work. Although the main cell culture conditions were standardized, slight differences were already observed when comparing the cytotoxicity of various concentrations of MP during the pilot studies performed to define suitable concentrations. Although increased LDH release was observed with concentrations of 100 μM MP and above in three of the four companies, no increased LDH leakage was observed by BA with concentrations up to 300 μM in a pilot experiment (Figure 1A). The reason for this was not investigated further, and concentrations that caused only marginal or no LDH release were chosen for the main experiment (20 and 100 μM).

Analysis of the gene expression data with one-dimensional hierarchical clustering using the whole set of genes available on the RG-U34A GeneChip revealed that the differences between laboratories were greater than the differences between treatment groups. This was not surprising, as it has already been observed in an interlaboratory analysis reported by Waring et al. (2004). However, when focusing on the statistically significant gene expression changes from the data sets of all laboratories (genes were obtained by using the statistical methods of laboratory SAG: *t*-test, *p* < 0.01, fold change > 1.5), the clustering results reflected the experimental design, allowing the high-concentration samples to be separated from the controls and low-dose samples (Figure 3B). In addition the hepatocyte cultures of BA and BI appeared to be more sensitive to MP treatment than those of RO and SAG because PCA showed the separation of the low dose from the untreated for BA and BI. This might be because RO and SAG perform a Percoll gradient to separate the live hepatocytes from dead cells. This also removes other cell types from the preparation and might affect the sensitivity of the test system. Thus, using a suitable statistical method, the effect of the treatment supersedes the experimental variability. Differences on the experimental systems such as cell preparation (Percoll purification step) were also detected. In addition to the statistical methods applied by SAG, RO used its own analysis method and cutoff values from all data sets to compare each of them with a reference *in vitro* toxicogenomics DB. This proprietary DB contained 17 known toxic compounds tested on rat hepatocytes, including an independent exposure to MP under slightly different experimental conditions. For three of the data sets (BA, BI, RO), the gene expression profiles allowed the correct identification of MP as the best match in the DB, independently of the site where the experiment was performed.

Next, we investigated the influence of the use of different data analysis strategies to identify altered genes on the same data set. The individual analysis methods are described in Table 2, including differences in the definition of cutoff values for parameters such as fold change or *p*-value. The arbitrary choice of these cutoff values is not trivial and greatly influences the outcome of the analysis. On the one hand, stringent cutoff values lead to a smaller false-positive rate and a high false-negative rate (or low power). This approach can be recommended if each single gene will be interpreted and discussed regarding safety assessment. However, important signals might be missed because relatively small changes in expression may be of high biologic and toxicologic relevance. On the other hand, less stringent filtering criteria cause a high number of false positives but ensure that no relevant genes will remain undetected. In our case, BA and SAG used stringent statistical approaches (*t*-test with *p*-value < 0.01, fold change > 1.5 fold), whereas BI and RO used smaller fold changes as cutoff criteria (1.2-fold or 1.25-fold, respectively). As expected, BA and SAG detected fewer regulated genes than did BI and RO (Figure 4, Table 2). For BI the obtained gene list was used as a first-pass filter for the comparison with in-house defined marker genes or for hypothesis generation with a subsequent in-depth analysis of selected genes. When all companies analyzed their own data with their own methods, only 14 probe sets were considered deregulated by all the users in all experiments, and an additional 103 were detected by three of the four laboratories (Figure 5). This demonstrates that an additional layer of complexity and a source of differing interpretation originate from different statistical analysis methods.

The gene changes observed after 24 hr of incubation with the test compound might not be ideal to elucidate the primary events (cause) that trigger the hepatotoxicity of MP. However, the elucidation of downstream gene expression changes, indicative of general cellular dysfunction as a consequence of MP toxicity is valuable as a possible predictor for hepatotoxicity. The identity of the genes that were found changed in at least three of four laboratories (117 genes) represent biologically relevant processes that are obviously affected by MP. Several genes involved in amino acid and nucleotide metabolism were down-regulated. Also, the expression of genes that play a role in the cell cycle and/or apoptosis was changed by MP. Among them, the mitogen-activated protein kinase 6 and ornithine decarboxylase antizyme inhibitor were up-regulated, whereas ectonucleotide pyrophosphatase/phosphodiesterase 2 and insulin growth factor–binding protein were down-regulated. These signals appear contradictory because those genes promoting cell proliferation are not regulated in the same direction. However, the detected changes were generally consistent across users, increasing the confidence in the findings. Another affected pathway involved genes related to the glutathione homeostasis. Ratra et al. (2000) showed that the levels of reduced glutathione are increased to 140% of the control after administration of MP to male Han:Wistar rats. In agreement with this, our experiments show that MP had a substantial effect on genes involved in glutathione metabolism (5-oxopro-linase) and glutathione conjugation (glutathione *S*-transferase 3 and Yb). Also, other genes involved in detoxification, such as l-gulono-gammalactone oxidase and sulfotranferase family 1A were down-regulated. MP also seems to have an effect on the energy balance of the liver. Many genes in the glycolysis pathway and several genes involved in mitochondrial function were down-regulated by the treatment. This finding is also in agreement with previous results obtained *in vivo* and *in vitro*. It has been described that MP leads to a significant increase in mitochondria of periportal hepatocytes in rats (Reznik-Schuller and Lijinski 1981). Also, MP caused mitochondrial dysfunction, as detected by mitochondrial swelling, significant losses of ATP, and loss of mitochondrial calcium homeostasis in cultured hepatocytes (Ratra et al. 1998). In addition to the metabolic and energy impairment responses, MP elicits a stress response in the hepatocytes. Reactive oxygen producing systems are repressed, and stress-response genes are up-regulated. This is indicative of the oxidative stress produced by MP (Ratra et al. 1998) and was also described using gene expression profiles of livers of rats treated with MP (Waring et al. 2004). We observed the up-regulation of the ribosome associated membrane protein 4, which belongs to a family consisting of several ribosome associated membrane protein sequences that are known to stabilize membrane proteins in response to stress (Yamaguchi et al. 1999). Also, the myeloid differentiation primary response gene 116 (*Gadd34*), whose overexpression promotes apoptosis (Hollander et al. 2003), was detected as induced. The Gadd family is known to be up-regulated upon cellular stress and was strongly up-regulated by MP after *in vivo* exposure (Waring et al. 2004). Because we analyzed the toxicity of MP in isolation, we cannot determine which of these gene changes are specific to MP or might be regulated by other compounds. Also, most of the gene-by-gene changes described occurred at the high concentration, concomitant with slight cytotoxicity. However, some of the differentially expressed genes were also detected at the low dose by some laboratories. It was clear from the clustering data that both RO and SAG could not separate the low dose from the untreated samples. Gene expression data from BA and BI, however, showed that > 25% of the genes were already detectable at the low concentration (Table 4). These two laboratories did not perform a Percoll purification step during the hepatocyte isolation procedure. This interesting finding led us to the hypothesis that in the presence of additional cell types not eliminated by a Percoll purification step (e.g., Kupffer cells or damaged hepatocytes), gene expression changes occur already at concentrations that do not show an effect on the viability of the cells. Further experiments with controlled cell compositions should be performed to clarify this point and define the best-suited *in vitro* system in terms of sensitivity.

Our results show that several factors from experimental conditions to statistical data analysis contribute to the interlaboratory variability observed for gene expression results. Our data and other published results (Harries et al. 2001; Waring et al. 2001) show that *in vitro* assays coupled with microarray analysis are useful for detection of hepatotoxicity and mechanistic elucidation of cellular events related to it. This applies best when the experimental and analytical variability is reduced to a minimum, which cannot always be ensured. However, we were able to show that using suitable statistical analysis tools, we could, despite the experimental variability, uncover the commonalities among the experiments. We demonstrated that using a subset of deregulated genes for the analysis, the effects of a high concentration of MP on the cells supersede the interlaboratory variability and that this variability does not mask clear treatment-dependent effects. This finding agrees with a similar analysis performed *in vivo* (Waring et al. 2004) and also held true when we compared the data obtained at several sites with one *in vitro* toxicogenomics DB. The encouraging outcome of the comparison with an independent DB is pivotal and indicates that gene expression profiles have the potential to be used as a diagnostic tool for toxicology. However, it is also clear from the presented results that the differences between laboratories make the gene-by-gene comparison of gene expression data from different sources very difficult. This task can be undertaken only with sound statistical tools that allow a relevant subset of genes to be selected.

From a mechanistic point of view, it is important to note that there was good concordance among all users regarding the affected biologic processes, as shown in Table 4. Most of the consistently regulated genes play a role in detoxification/metabolism, processes of growth and death control, immune response, stress, and transport. This indicates that the interpretation of the data from different sources leads to similar conclusions in terms of toxicity and underlying mechanisms despite the differences in number and identity of genes and in the intensity of the regulation.

In summary, our data show that both experimental and statistical variability are important sources of different outcomes between laboratories. To minimize the experimental variation, it is advisable to perform the cell culture and microarray experiments whenever possible at the same experimental site. This is not always possible because often experimental protocols need to be transferable. In these cases, suitable and robust statistical analyses help overcome the differences. Also, we showed that cellular mechanisms involved in MP toxicity can be consistently detected, as illustrated by the gene expression changes listed in Table 4. In addition the positive outcome of the comparison with an *in vitro* DB underlines that microarray analyses of *in vitro* systems are robust and can be predictive of toxicity. Whether the involved cellular pathways are specific for MP and are causal to the toxicity *in vitro* and/or *in vivo* requires further investigations.

## Figures and Tables

**Table 1:** Sample preparation methods and data analysis tools used by the contributing companies.

Analysis site	Viability (%)^a^	Cell purification	LDH assay	RNA extraction	IVT	Data condensation/normalization	Data analysis tools/software
BA	85, 90, 89	None	Hitachi 717/Roche	RNeasy	Enzo-Affymetrix	MAS 5.0	Expressionist
BI	81, 74, 73	None	Hitachi 917/Roche	RNeasy (+ Prot. K)	Enzo-Affymetrix	MAS 5.0	SAS
RO	87, 92, 90	Percoll	ADVIA 1650/LDH P-L Bayer	RNAzol/Bio 101	Ambion	MAS 5.0	In-house software
SAG	84, 84, 72	Percoll	Hitachi/SYS1 Roche	RNeasy	Enzo-Affymetrix	In-house software	Expressionist

Abbreviations: Enzo-Affymetrix, Enzo Diagnostics Inc. and Affymetrix, Inc.; IVT, *in vitro* transcription; Prot. K, proteinase K.

a Cell viability of the hepatocyte preparations in the main study (*n* = 3).

**Table 2:** Number of genes regulated by MP of the BI experiments calculated by the four different laboratories according to their applied method.

Treatment	BA	BI	RO	SAG
Low-dose	75	1,296	687	84
High-dose	211	1,914	1,011	289
Union	256	2,486	1,286	332
Method	MAS 5.0 Welch’s *t*-test *p* < 0.01	MAS 5.0 Mann-Whitney *U*-test *p* < 0.05	MAS 5.0 Paired *t*-test, *p* < 0.1	In-house software *t*-test *p* < 0.01
Cutoff	> 1.5-fold change	> 1.2-fold change	> 1.25-fold change	> 1.5-fold change

**Table 3:** Intersections between the genes regulated by MP per laboratory calculated by its own methods (values in brackets are percentage of that company’s genes shared with the respective other companies).

Company	BA (%)	BI (%)	RO (%)	SAG (%)
BA	185	138 (6)	84 (8)	16 (13)
BI	138 (75)	2,486	579 (53)	82 (65)
RO	84 (45)	579 (23)	1,085	74 (59)
SAG	16 (9)	82 (3)	74 (7)	126

**Table 4:** Genes regulated by a low or high dose of MP detected by at least three of the four laboratories.

		BA		BI		RO		SAG		
Affymetrix probe set ID^a^	Gene description^a^	20 μM	100 μM	20 μM	100 μM	20 μM	100 μM	20 μM	100 μM	Direction of change
Amino acid metabolism										
AB003400_at	d-amino acid oxidase	−1.29	−2.13^b^	−1.96^b^	−3.35^b^	−1.22	−1.89^b^	−1.06	−1.52	Down
AF038870_at	betaine-homocysteine methyltransferase	−2.28^b^	−5.02^b^	−4.41^b^	−7.21^b^	−1.23	−2.04^b^	1.20	−1.62	Down
D17370_at	CTL target antigen	−1.27	−2.32^b^	−1.50^b^	−2.58^b^	1.17	−1.43^b^	1.04	−1.57	Down
D87839_g_at	4-aminobutyrate aminotransferase	−2.22^b^	−4.81^b^	−2.34^b^	−8.16^b^	−1.54^b^	−5.00^b^	−1.09	−2.64^b^	Down
J02827_g_at	branched chain alpha-ketoacid dehydrogenase subunit E1 alpha	−1.54	−1.45	−1.46^b^	−1.59^b^	−1.18	−1.35^b^	1.03	−1.80^b^	Down
M88347_s_at	cystathionine beta synthase	−1.50	−2.44^b^	−1.60^b^	−2.81^b^	−1.23	−2.44^b^	−1.29	−1.29	Down
U68168_at	kynureninase (L-kynurenine hydrolase)	−2.12^b^	−5.46^b^	−2.36^b^	−6.22^b^	−1.28^b^	−4.17^b^	−1.08	−2.30^b^	Down
Cell-cycle/apoptosis										
AB002086_at	p47 protein	1.21	1.35	1.37^b^	1.56^b^	1.26^b^	1.60^b^	1.23	1.66^b^	Up
AF020618_g_at	myeloid differentiation primary response gene 116	2.05^b^	4.71^b^	1.82^b^	5.25^b^	1.21	3.88^b^	−1.06	2.55^b^	Up
D28560_at	ectonucleotide pyrophosphatase/phosphodiesterase 2	−1.22	−2.01^b^	−1.65^b^	−3.24^b^	−1.22	−2.04^b^	−1.15	−2.28	Down
D28560_g_at	ectonucleotide pyrophosphatase/phosphodiesterase 2	−1.67^b^	−2.43^b^	−3.11^b^	−9.83^b^	−1.28^b^	−2.27^b^	−1.24	−2.11	Down
rc_AI043631_s_at	ornithine decarboxylase antizyme inhibitor	1.67^b^	2.75^b^	2.72^b^	4.25^b^	1.29	2.90^b^	−1.04	2.26	Up
S46785_at	insulin-like growth factor binding protein complex acid-labile subunit	1.03	−1.10	−1.41^b^	−1.69^b^	−1.20	−1.79^b^	1.67^b^	1.00	Down
Detoxification										
D14564cds_s_at	L-gulono-gamma-lactone oxidase (BLAST)	−1.32	−1.49	−2.05^b^	−2.50^b^	−1.20	−2.17^b^	−1.04	−1.78^b^	Down
J03914cds_s_at	glutathione *S*-transferase Yb subunit gene	−1.39	−1.92^b^	−1.43^b^	−1.93^b^	−1.14	−1.41^b^	−1.01	−1.66^b^	Down
L19998_at	sulfotransferase family 1A, phenol-preferring, member 1	−1.93	−4.14	−5.86^b^	−9.35^b^	−1.59^b^	−8.33^b^	−1.21	−6.39^b^	Down
L19998_g_at	sulfotransferase family 1A, phenol-preferring, member 1	−1.71	−3.36	−5.97^b^	−6.94^b^	−1.59^b^	−6.25^b^	−1.17	−5.14^b^	Down
M23601_at	monoamine oxidase B	−1.45	−2.40^b^	−2.07^b^	−4.65^b^	−1.06	−2.44^b^	−1.04	−2.08^b^	Down
rc_AA892234_at	ESTs, highly similar to microsomal GST 3	−1.46	−2.26^b^	−1.55^b^	−3.54^b^	−1.33^b^	−2.33^b^	−1.02	−2.18	Down
U70825_at	5-oxoprolinase (ATP-hydrolyzing)	−1.47	−2.30^b^	−1.95^b^	−5.46^b^	−1.23	−3.33^b^	−1.14	−2.05	Down
Glycolysis and gluconeogenesis										
AF062740_at	pyruvate dehydrogenase phosphatase isoenzyme 1	1.30	1. 69^b^	−1.42^b^	1.37^b^	−1.04	1.31^b^	1.32	1.39	Up
J05446_at	glycogen synthase 2 (liver)	−2.00^b^	−3.33^b^	−2.35^b^	−5.92^b^	−1.19	−2.86^b^	1.05	−2.11	Down
M12919mRNA#2_at	aldolase A	1.25	1.55	1.30^b^	1.84^b^	−1.01	1.76^b^	1.11	1.72^b^	Up
M83298_at	protein phosphatase 2, regulatory subunit B, α isoform	1.37	1.78^b^	1.61^b^	2.18^b^	1.09	1.46^b^	−1.07	1.32	Up
M86240_at	fructose-1,6-bisphosphatase 1	−2.03^b^	−2.55^b^	−2.14^b^	−3.15^b^	−1.24	−2.70^b^	−1.02	−2.32^b^	Down
rc_AA892395_s_at	aldolase B	−2.12	−3.59	−1.73^b^	−4.84^b^	−1.09	−2.78^b^	1.01	−2.37^b^	Down
rc_AA945442_at	glucokinase regulatory protein	−1.67^b^	−2.06^b^	−1.38^b^	−2.02^b^	−1.30	−1.61^b^	−1.18	−1.88^b^	Down
S79213_at	phosphatase inhibitor-2	1.57^b^	2.11^b^	2.00^b^	2.48^b^	1.20	1.39^b^	−1.09	1.30	Up
U32314_g_at	pyruvate carboxylase	−1.55^b^	−1.62^b^	−2.28^b^	−4.61^b^	−1.06	−1.52^b^	−1.08	−1.30	Down
X02291exon_s_at	aldolase B (BLAST)	−1.58	−2.24	−1.39^b^	−3.00^b^	−1.08	−2.17^b^	−1.06	−2.03^b^	Down
X53428cds_s_at	glycogen synthase kinase 3 beta	1.52^b^	1.82^b^	2.12^b^	2.78^b^	1.08	2.41^b^	1.01	2.60	Up
X73653_at	glycogen synthase kinase 3 beta	1.32	1.67^b^	1.99	2.42	1.03	1.87^b^	1.09	2.72^b^	Up
Immune response										
AF029240_g_at	BM1k MHC class Ib antigen, strain SHR	−1.61	−1.54	−1.63^b^	−2.42^b^	−1.20	−1.82^b^	−1.18	−2.09^b^	Down
L12025_at	tumor-associated glycoprotein pE4	2.15^b^	3.43^b^	2.59^b^	5.30^b^	1.19	3.03^b^	−1.02	1.81	Up
U47031_at	purinergic receptor P2X, ligand-gated ion channel	−1.14	−1.13	−1.21^b^	−1.36^b^	−1.14	−1.56^b^	−1.09	−1.62^b^	Down
Mitochondrial function										
AF062740_at	pyruvate dehydrogenase phosphatase isoenzyme 1	1.30	1.69^b^	−1.42	1.37^b^	−1.04	1.31^b^	1.32	1.39	Up
D00569_g_at	2,4-dienoyl CoA reductase 1, mitochondrial	−1.39	−1.63^b^	−1.11	−2.07^b^	−1.18	−1.64^b^	−1.05	−1.42	Down
D30740_at	14-3-3 protein mRNA for mitochondrial import stimulation factor (MSF) S1 subunit	1.32	1.51^b^	1.56^b^	1.78^b^	1.17	1.29^b^	1.08	1.30	Up
J05029_s_at	acyl coenzyme A dehydrogenase, long chain	−1.02	−1.25	−1.43^b^	−1.98^b^	−1.09	−1.79^b^	−1.08	−1.54^b^	Down
J05030_at	acyl coenzyme A dehydrogenase, short chain	−1.27	−1.54^b^	−1.62^b^	−1.63^b^	−1.10	−1.82^b^	1.01	−1.58	Down
M23601_at	monoamine oxidase B	−1.45	−2.40^b^	−2.07^b^	−4.65^b^	−1.06	−2.44^b^	1.04	−2.08^b^	Down
M33648_at	mitochondrial 3-hydroxy-3-methylglutaryl-CoA synthase	−4.16^b^	−11.38^b^	−2.92^b^	−13.51^b^	−1.30	−6.25^b^	−1.06	−2.93^b^	Down
M33648_g_at	mitochondrial 3-hydroxy-3-methylglutaryl-CoA synthase	−2.40^b^	−7.85^b^	−2.46^b^	−10.44^b^	−1.25^b^	−4.55^b^	1.00	−2.02	Down
rc_AA817846_at	ESTs, highly similar to BDH_RAT d-beta-hydroxybutyrate dehydrogenase	−1.95	−3.25	−3.32^b^	−8.04^b^	−1.41	−5.00^b^	−1.07	−2.58^b^	Down
rc_AI176422_at	ESTs, highly similar to S41115 probable flavoprotein-ubiquinone oxidoreductase	−1.22	−1.53	−1.61^b^	−2.36^b^	−1.19	−1.30^b^	−1.03	−1.79^b^	Down
U32314_g_at	pyruvate carboxylase	−1.55	−1.62	−2.28^b^	−4.61^b^	−1.06	−1.52^b^	−1.09	−1.30	Down
Y12635_at	ATPase, H^+^ transporting, lysosomal, isoform 2	1.40	2.43^b^	1.51^b^	2.74^b^	1.15	2.18^b^	1.04	1.81	Up
Nucleotide metabolism										
D28560_at	ectonucleotide pyrophosphatase/phosphodiesterase 2	−1.22	−2.01^b^	−1.65^b^	−3.24^b^	−1.22	−2.04^b^	−1.15	−2.28	Down
D28560_g_at	ectonucleotide pyrophosphatase/phosphodiesterase 2	−1.67^b^	−2.43^b^	−3.11^b^	−9.83^b^	−1.28^b^	−2.27^b^	−1.24	−2.11	Down
M97662_at	ureidopropionase, beta	−1.93	−3.08	−2.99^b^	−4.56^b^	−1.43^b^	−3.13^b^	−1.10	−4.08^b^	Down
rc_AA799402_at	ESTs, weakly similar to S18140 hypoxanthine phosphoribosyl-transferase	−1.10	−1.93	−1.79^b^	−1.67^b^	−1.27	−1.27^b^	−1.11	−1.59^b^	Down
Protein metabolism										
AF100470_g_at	ribosome associated membrane protein 4	1.11	1.30	1.29^b^	1.41^b^	1.18	1.61^b^	1.07	1.57^b^	Up
L38482_g_at	serine protease gene	1.11	1.29	1.20^b^	1.09	1.11	1.40^b^	1.09	1.78^b^	Up
M96633_at	mitochondrial intermediate peptidase	−1.48	−2.45^b^	−1.74^b^	−3.47^b^	−1.23	−2.22^b^	1.08	−1.77	Down
rc_AA892831_s_at	ESTs, highly similar to JC6524 26S proteasome regulatory complex chain p44.5	1.12	1.28	1.44^b^	1.32^b^	1.09	1.50^b^	1.05	1.84^b^	Up
X70900_at	hepsin	−1.59^b^	−2.45^b^	−1.69^b^	−2.56^b^	−1.23	−1.96^b^	1.02	−2.56^b^	Down
Signal transduction										
AF036537_g_at	homocysteine respondent protein HCYP2	1.56	1.67	1.83^b^	1.85^b^	1.38^b^	2.18^b^	−1.16	1.95^b^	Up
AF076619_at	growth factor receptor bound protein 14	−1.11	−1.67^b^	−1.09	−2.23^b^	−1.08	−1.79^b^	−1.03	−1.35	Down
L14323_at	phospholipase C-beta1	−1.27	−2.03^b^	−1.55^b^	−3.34^b^	1.06	1.67^b^	−1.05	−1.33	Down
M64301_g_at	mitogen-activated protein kinase 6	1.26	2.17^b^	1.06	2.51^b^	−1.06	1.62^b^	−1.04	1.27	Up
M83298_at	protein phosphatase 2 (formerly 2A), regulatory subunitB (PR 52), alpha isoform	1.37	1.78^b^	1.61^b^	2.18^b^	1.09	1.46^b^	−1.07	1.32	Up
rc_AA891580_at	ESTs, highly similar to cylindromatosis (turban tumor syndrome); cylindromatosis 1	1.27	1.61^b^	1.90^b^	2.02^b^	1.27^b^	1.32	1.00	1.30	Up
rc_AI070721_s_at	glial cell line derived neurotrophic factor family receptor α1	−2.49	−2.68	−1.19	−2.15^b^	−1.32	−2.77^b^	−1.07	−1.95^b^	Down
rc_AI171630_s_at	p38 mitogen activated protein kinase	−1.21	−1.70^b^	−1.75^b^	−2.37^b^	−1.23	−1.64^b^	−1.10	−1.29	Down
Stress response										
M23601_at	monoamine oxidase B	−1.45	−2.40^b^	−2.07^b^	−4.65^b^	−1.06	−2.44^b^	−1.04	−2.08^b^	Down
M86389cds_s_at	heat shock 27 kDa protein	1.65	2.15	3.11^b^	2.24^b^	1.16	2.22^b^	1.25	2.87^b^	Up
rc_AA891286_at	thioredoxin reductase 1	1.45	1.78^b^	1.77^b^	1.85^b^	1.22	1.66^b^	1.12	1.40	Up
rc_AI171630_s_at	p38 mitogen activated protein kinase	−1.21	−1.70^b^	−1.75^b^	−2.37^b^	−1.23	−1.64^b^	−1.10	−1.29	Down
rc_AI179610_at	heme oxygenase	1.34	3.37^b^	1.87	3.45	1.18	2.99^b^	1.12	2.74^b^	Up
Transcription										
AB012230_at	NF1-B1	−1.20	−1.86^b^	−1.03	−2.11^b^	−1.32^b^	−1.32	1.00	1.00	Down
AF003926_at	nuclear receptor subfamily 2, group F, member 6	−1.24	−1.69^b^	−1.03	−1.58^b^	−1.10	−1.41^b^	−1.04	−1.41	Down
AF016387_g_at	retinoid X receptor gamma	−1.31	−2.15^b^	−1.39^b^	−2.56^b^	−1.10	−1.69^b^	1.00	1.00	Down
Transport										
AB015433_s_at	solute carrier family 3, member 2	1.36	1.89^b^	1.96^b^	3.30^b^	1.18	1.77^b^	1.07	1.89	Up
U72741_g_at	lectin, galactose binding, soluble 9 (galectin-9)	−1.37	−1.46	−2.28^b^	−3.44^b^	−1.23	−1.49^b^	−1.06	−1.98^b^	Down
Z36944cds_at	putative chloride channel (similar to Mm Clcn4-2)	−1.88^b^	−2.50^b^	−1.57^b^	−1.93^b^	−1.12	−2.13^b^	−1.09	−1.37	Down

a From Affymetrix, Inc. (http://www.affymetrix.com).

b Significant fold changes.

**Table 5:** Comparisons with the Roche *in vitro* toxicogenomics database.

			Similarity index							
			BA		BI		RO		SAG	
Data set	Dose (μM)	Mechanism	High	Low	High	Low	High	Low	High	Low
MP_BA_high	100	Direct reaction	N/A	28.55^a^	28.44^a^	24.23^a^	24.06^a^	4.72	17.78	0.31
MP_BA_low	20	Direct reaction	28.55^a^	N/A	12.66	18.46	11.78	4.95	15.00	0.35
MP_BI_high	100	Direct reaction	28.24^a^	12.66	N/A	33.44^a^	26.02^a^	3.12	13.97	0.00
MP_BI_low	20	Direct reaction	24.23	18.46^a^	33.44^a^	N/A	21.67	5.51	21.14^a^	0.84
MP_RO_high	100	Direct reaction	24.06	11.78	26.02	21.67	N/A	4.95	20.29^a^	1.10
MP_RO_low	20	Direct reaction	4.72	4.95	3.12	5.51	6.69	N/A	6.69	1.06
MP_SAG_high	100	Direct reaction	17.78	15.00	13.97	21.14	20.29	6.69^a^	N/A	3.77^a^
MP_SAG_low	20	Direct reaction	0.31	0.35	0.00	0.84	1.10	1.06	3.77	N/A
MP_DB_100	100	Direct reaction	22.16^b^	12.81^b^	20.95^b^	19.50^b^	21.82^b^	6.69^a^^b^	15.71^b^	0.94
MP_DB_300	300	Direct reaction	20.08^b^	8.47	23.21^b^	12.25	17.51^b^	3.08	7.60	0.41
Other_cmp	N/A	Direct reaction	2.82	3.74	2.80	4.88	2.57	4.16	4.25	2.83^a^^b^
Other_cmp	N/A	Direct reaction	11.98	8.45	12.65	13.39	11.87	4.35^b^	11.21	1.76^b^
Other_cmp	N/A	Direct reaction	17.93	11.84^b^	16.3	14.35^b^	14.33	3.84	10.92	1.14
Other_cmp	N/A	Perox. prolif.	< 0	< 0	< 0	< 0	< 0	< 0	0.85	0.00
Other_cmp	N/A	Perox. prolif.	< 0	< 0	< 0	< 0	< 0	0.27	0.72	< 0

Abbreviations: N/A, not applicable; Other_cmp: other compound in DB; Perox. prolif., peroxisome proliferators.

a Top two of comparison including data sets of this study.

b Top two of comparison without data sets of this study.
